# The organization of melanopsin-immunoreactive cells in microbat retina

**DOI:** 10.1371/journal.pone.0190435

**Published:** 2018-01-05

**Authors:** Mi-Jin Jeong, Hang-Gu Kim, Chang-Jin Jeon

**Affiliations:** Department of Biology, School of Life Sciences, BK21 Plus KNU Creative BioResearch Group, College of Natural Sciences, and Brain Science and Engineering Institute, Kyungpook National University, Daegu, South Korea; National Eye Centre, UNITED STATES

## Abstract

Intrinsically photosensitive retinal ganglion cells (ipRGCs) respond to light and play roles in non-image forming vision, such as circadian rhythms, pupil responses, and sleep regulation, or image forming vision, such as processing visual information and directing eye movements in response to visual clues. The purpose of the present study was to identify the distribution, types, and proportion of melanopsin-immunoreactive (IR) cells in the retina of a nocturnal animal, i.e., the microbat (*Rhinolophus ferrumequinum*). Three types of melanopsin-IR cells were observed in the present study. The M1 type had dendritic arbors that extended into the OFF sublayer of the inner plexiform layer (IPL). M1 soma locations were identified either in the ganglion cell layer (GCL, M1c; 21.00%) or in the inner nuclear layer (INL, M1d; 5.15%). The M2 type had monostratified dendrites in the ON sublayer of the IPL and their cell bodies lay in the GCL (M2; 5.79%). The M3 type was bistratified cells with dendrites in both the ON and OFF sublayers of the IPL. M3 soma locations were either in the GCL (M3c; 26.66%) or INL (M3d; 4.69%). Additionally, some M3c cells had curved dendrites leading up towards the OFF sublayer of the IPL and down to the ON sublayer of the IPL (M3c-crv; 7.67%). Melanopsin-IR cells displayed a medium soma size and medium dendritic field diameters. There were 2–5 primary dendrites and sparsely branched dendrites with varicosities. The total number of the neurons in the GCL was 12,254.17 ± 660.39 and that of the optic nerve axons was 5,179.04 ± 208.00 in the *R*. *ferrumequinum* retina. The total number of melanopsin-IR cells was 819.74 ± 52.03. The ipRGCs constituted approximately 15.83% of the total RGC population. This study demonstrated that the nocturnal microbat, *R*. *ferrumequinum*, has a much higher density of melanopsin-IR cells than documented in diurnal animals.

## Introduction

The intrinsically photosensitive retinal ganglion cells (ipRGCs) are retinal ganglion cells (RGCs) that are considered a third class of photoreceptors, which respond to light. These ipRGCs have been studied for more than a decade and several of their anatomical and functional characteristics have been revealed [1–5]. These retinal cells innervate many brain areas, including the suprachiasmatic nucleus (SCN), ventral subparaventricular zone (vSPZ), intergeniculate leaflet (IGL), pretectal area (PTA), olivary pretectal nucleus (OPN), and ventrolateral preoptic nucleus (VLPO). These areas contribute to non-image forming responses to light, which include the regulation of circadian rhythm, pupil constriction, and sleep regulation [6–12]. In addition, recent studies demonstrated that ipRGCs also contribute to vision projecting to vision-related areas such as the lateral geniculate nucleus (LGN) and superior colliculus (SC) [13–16].

ipRGCs comprise a very minor portion of RGCs in mammals. It comprises from less than 0.2% in marmosets [17] to 2.5% in rats [18]. At least five subtypes of ipRGCs have been identified in the mammalian retina, based on cell body location and dendritic stratification [3, 19]. The M1 type has a cell body in the ganglion cell layer (GCL) and monostratified dendrites stretching toward the OFF sublayer of the inner plexiform layer (IPL) [20, 21]. The M2 type has a cell body in the GCL and monostratified dendrites stretching toward the ON sublayer of the IPL [10, 22]. The M3 type also has a cell body in the GCL, but its distinguishing feature is the bistratified dendrites that stretch toward both the ON and OFF sublayers of the IPL [20, 23]. The M4 and M5 types appear to be like the M2 type, with a cell body in the GCL and dendrites stretching toward the ON sublayer of the IPL. However, the M4 type has the largest cell body of all the subtypes of ipRGCs, as well as a larger dendritic field size and more complex dendritic arbors than the M2 type. In contrast, the M5 type has a smaller cell body and dendritic field size, but more highly branched arbors than the M2 type [15, 24].

Bats are the second largest order of mammal and present throughout most of the world. They can be divided into two main groups as follows: *Megachiroptera* (megabats) and *Microchiroptera* (microbats). Megabats have not only large eyes but also well-organized visual cortex, and advanced visual organizations [25, 26]. Microbats, however, have very tiny eyes and depend on echolocation for navigation and finding prey [27, 28]. Thus, it was postulated that microbats have poorly developed visual processes in the retina. However, previous studies demonstrated that microbats have developed retinofugal projections and a well-organized retina, which includes general retinal elements for visual processing [29–37]. These elements include rod and cone bipolar cells, AII amacrine cells, and RGCs [29, 34, 36]. In addition, several studies revealed the existence of rod and cone photoreceptors in microbat retina [29, 30, 33, 34]. However, the existence of the third photoreceptor, ipRGCs, has not yet been identified in any bats.

In the present study, we aimed to identify the presence of ipRGCs in the bat retina. Since ipRGCs play a key role in regulating the circadian rhythm, we also evaluated differences in the population of ipRGCs between diurnal and nocturnal animals with long sleeping habits. The greater horseshoe bat (*Rhinolophus ferrumequinum*), which is insectivorous, cave-dwelling, and a typically nocturnal microbat species, was used in the current study. Our results demonstrated three types of ipRGCs in the *R*. *ferrumequinum* retina, i.e., the M1 type, M2 type, and M3 type cells, which surprisingly accounted for 15.83% of total RGCs.

## Materials and methods

### Animals and tissue preparation

Adult greater horseshoe bats (*Rhinolophus ferrumequinum*) were used in the current study. All microbats were captured from a cave in the district of Milyang, South Korea. The microbats were anesthetized with a mixture containing ketamine hydrochloride (30–40 mg/kg) and xylazine (3–6 mg/kg). Proparacaine HCl (100–200 μl) was used to suppress the blink reflex of the cornea. The bat eyes were then instantly enucleated and the retinas were isolated. Then, the animals were sacrificed using an overdose of the same anesthetics. The isolated retinas were fixed for 2 h at 4°C in a buffer containing 4% paraformaldehyde in 0.1 M phosphate buffer (PB, pH 7.4). The retinas were processed as whole-mounts, and cut into 50 μm thick sections using a vibratome. All the investigations that animals are involved, conformed to the guidelines of the ARVO Statement for the Use of Animals in Ophthalmic and Vision Research. This research was also approved (permission no. 2015-0082-2) by the animal rights committee at Kyungpook National University, Daegu, South Korea.

### Fluorescence immunocytochemistry

Polyclonal rabbit anti-melanopsin (1:200, Thermo Scientific, Rockford, IL, USA) was used as the primary antibody. Cy3-conjugated goat anti-rabbit immunoglobulin IgG (1:200, Vector Laboratories, Burlingame, CA, USA) was used as the secondary antibody. Retinal tissues were processed as whole-mounts and cut into 50 μm thick vertical sections using a vibratome. We used the standard immunocytochemical techniques, and methods, which are detailed in our previous report [38]. In the vertical sections, the tissue was counterstained with 4', 6'-diamidino-2-phenylindole (DAPI) (Molecular Probes, Eugene, OR, USA). The tissue was coverslipped using Vectashield^®^ mounting medium (Vector Laboratories, Burlingame, CA, USA). Images were obtained using a Zeiss LSM 700 laser scanning confocal microscope (Carl Zeiss Meditec, Inc., Jena, Germany).

### Specificity of primary antibody

**Fig 1:**
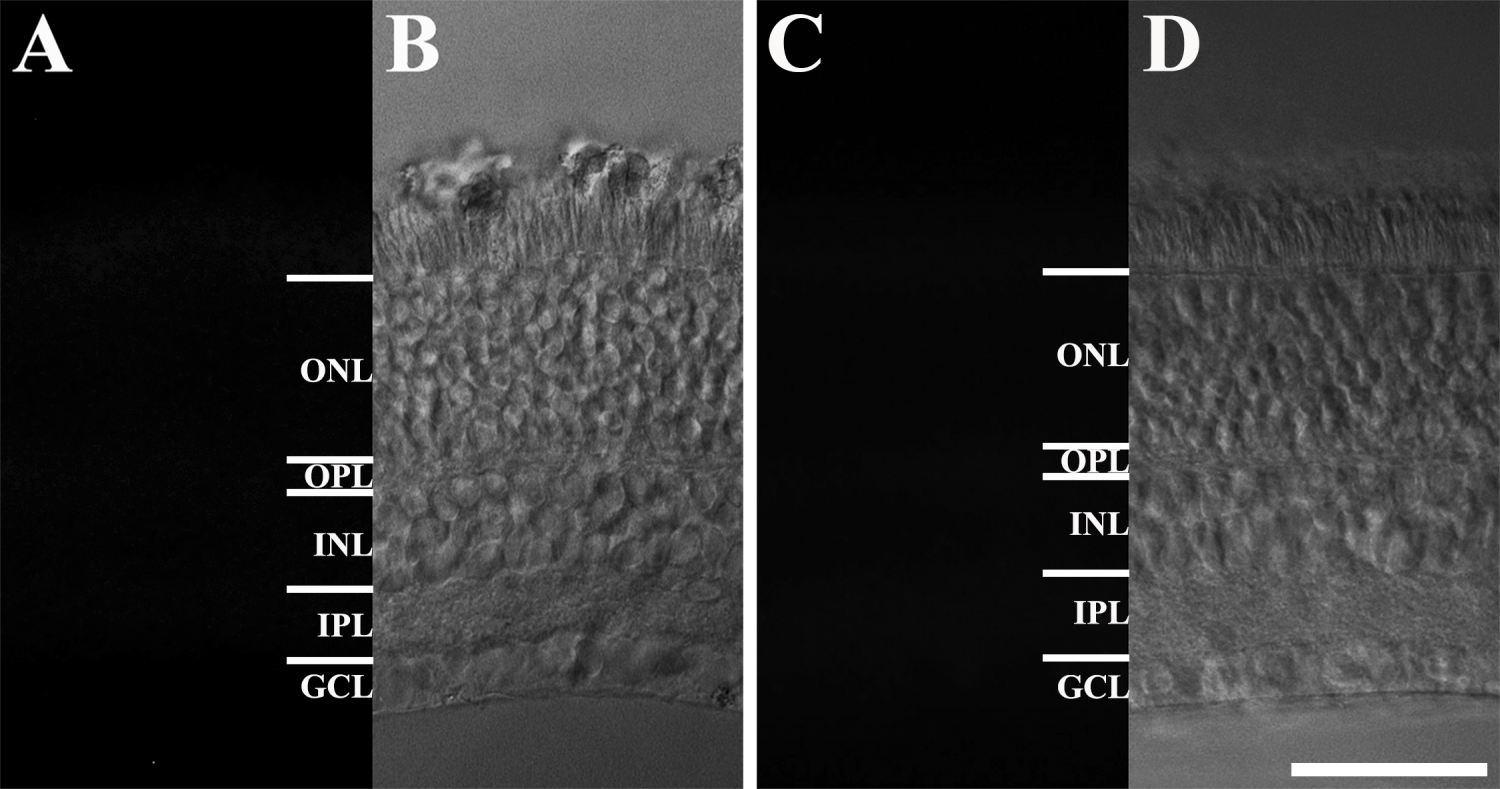
Negative and preabsorption tests in the *R*. *ferrumequinum* retina. (A, B) The negative test. (C, D) The preabsorption test. These tests were performed to assess the specificity of the rabbit polyclonal melanopsin antibody in the *R*. *ferrumequinum* retina. Melanopsin-IR cells were not detected in the *R*. *ferrumequinum* retina. IR, immunoreactive; GCL, ganglion cell layer; IPL, inner plexiform layer; INL, inner nuclear layer; OPL, outer plexiform layer; ONL, outer nuclear layer. Scale bar = 50 μm.

A rabbit polyclonal melanopsin antibody was obtained from Thermo Scientific (Cat# PA1-781, Rockford, IL, USA). This antibody was raised against a C-terminal peptide from rat melanopsin (E (a.a. 455) Q K S K T P K T K R H L P S L D R R M (a.a. 474)) and could detect melanopsin in RGCs. The specificity of this antibody to melanopsin-IR cells has been previously demonstrated in the mouse [6, 39, 40], rat [41], and even in humans [42]. To assess the specificity of the rabbit melanopsin antibody, we carried out two tests as follows: a negative control test and preabsorption test. The negative control test was performed under the same experimental conditions, except without the inclusion of the primary antibody. For the preabsorption test, anti-melanopsin blocking peptide (Peptron, Daejeon, South Korea) was mixed with the primary antibody at a 10:1 ratio to inactivate the primary antibody. The mixture was then pre-incubated at room temperature for 1 h. The tissues were incubated with the preabsorbed antibody, in place of the primary antibody. The negative control and preabsorption tests revealed no labeling in the *R*. *ferrumequinum* retina (Fig 1).

### Quantitative analysis

The soma and dendritic field diameters were determined using a digital camera (Zeiss AxioCam HRc; AxioVision 4; Zeiss, Welwyn Garden City, UK). The dendritic fields were in the mid-peripheral retina. We selected three retinas with the best labeling and measured the soma diameter of 180 cells (120 cells for general soma diameter and 60 cells for soma diameter by cell types) and the dendritic field diameter of 120 cells (60 cells for general dendritic field diameter and 60 cells for dendritic field diameter and dendritic length by cell types). The soma diameter of the melanopsin-IR cells was assessed using a Zeiss Axioplan microscope with a 40× Zeiss Plan-Apochromat objective (Carl Zeiss). The soma was circled with a pen on the monitor. The dendritic field diameters were also assessed using a 40× Zeiss Plan-Apochromat objective (Carl Zeiss) by connecting the distal-most tips of the dendrites and measuring the diameter. The total dendritic lengths were measured using image J to trace total dendrites of the neuron.

Whole-mount drawings of the melanopsin-IR cells were produced using a Zeiss Axioplan microscope (Carl Zeiss), with a 40× Zeiss Plan-Apochromat objective (Carl Zeiss). Melanopsin-IR cells were imaged on the computer monitor and cells were drawn on acetate sheets. The final images were drawn using Adobe Photoshop CS4 (Adobe Systems, San Jose, CA, USA). Based on stratifications, the colors were used differently. Dendrites were drawn in blue (the ON sublayer of the IPL) and red (the OFF sublayer of the IPL), while the cell bodies were drawn in black (GCL) and green (INL) on the acetate sheets. The final color picture was generated by superimposing the acetate sheets onto drawing paper.

For cell counts, all imaging was performed on a digital camera (Zeiss AxioCam HRc; (AxioVision 4; Zeiss, Welwyn Garden City, UK), with a 40× Zeiss Plan-Apochromat objective (Carl Zeiss). We identified four whole-mount retinas where the fluorescence was clearest and used the selected retinas to assess the density of melanopsin-IR cells. In two of the retinas, we sampled 16 areas (with one sample area representing 310 × 310 μm^2^). The sample areas were selected from evenly distributed positions across the retina. We then counted the number of melanopsin-IR cells along the central dorsoventral and nasotemporal axes. In the other two whole-mount retinas, all the melanopsin-IR cells were counted in 37 sampled areas.

The cell types were identified using three whole-mount retinas that displayed optimal fluorescence. Images were obtained using a Zeiss LSM 700 laser scanning confocal microscope with a 40× objective (Carl Zeiss). Serial optical sections (22–32 images/field, 1 μm thick) were imaged and 11 sequential fields (one sample area was 250 × 300 μm^2^) were sampled in the mid-peripheral regions of the three retinas. To analyze melanopsin-IR cell types, cells were drawn onto acetate sheeting and each serial optical section was assessed. The colors were used differently based on stratifications as follows: black was used for the GCL, blue for the ON sublayer of the IPL, red for the OFF sublayer of the IPL, and green for the INL.

Statistical analyses were performed using SPSS 11.5 (SPSS Inc., Chicago, IL, USA). Statistical comparison of means (for the M1, M2 and M3 types) was performed via a one-way analysis of variation (ANOVA), with the least significant difference (LSD) multiple-range test. Data were considered significant at p < 0.05 and expressed as mean ± standard deviation (SD).

### Electron microscopy of the optic nerve and total ganglion cell counts

The bats (n = 3) were anesthetized and transcardially perfused with a mixture of 4% paraformaldehyde and 0.3% glutaraldehyde in 0.1 M phosphate buffer (PB, pH 7.4). The optic nerves were carefully dissected and fixed for 2 h in 2.5% glutaraldehyde, then post-fixed for 90 mins in 1% osmium tetroxide in 0.05 M phosphate buffer (PB, pH 7.4). The nerves were dehydrated in ethanol, and embedded in Epon Araldite. Semi-thin sections (i.e., with a thickness of 1–2 μm) were obtained from the optic nerve to identify the axons. Slides were stained with 0.1% toluidine blue in 1% sodium borate for 20 min at room temperature. They were then coverslipped with Permount Mounting Medium (Fisher scientific, Pittsburgh, PA, USA). Images were obtained using a Zeiss AxioCam HRc digital camera (AxioVision 4; Zeiss, Welwyn Garden City, UK). Ultrathin sections (~80 nm thick) were cut perpendicularly to the long axis of the nerve on an MT-X ultramicrotome (RMC Co., CA, USA), and collected on 200 mesh copper grids. Nerve sections were obtained at ~300 μm from the posterior pole of the eye, where most of the fibers are myelinated. Sections were counterstained with uranyl acetate and lead citrate. Images were viewed on an H-7600 electron microscope (HITACHI, LTD, Tokyo, Japan). At 700× nominal magnification, six rectangular images (with a sampled area of 726.16 μm^2^ per image) were randomly selected from each optic nerve section. The ultrastructure of the optic nerve was visualized with the TEM at 6,680× magnification. This confirmed that most of the axons were myelinated. The total number of axons was counted by drawing the axons onto acetate paper. Approximately 220–310 axons were identified on each image. Toluidine blue staining was performed to identify the total area of the optic nerve. The samples were imaged with the Zeiss AxioCam HR and the total area of the optic nerve was approximately 12,700–13,800 μm^2^. The total number of axons in the optic nerve was calculated by multiplying the density of axons in sampled area by the total optic nerve area. Therefore, we calculated that approximately 5,180 axons were present in the optic nerve.

In order to calculate the fraction of cells in the GCL that were ganglion cells, we counted the total number of cells in this layer of the retina from the three microbats, *R*. *ferrumequinum*. After fixation, the retinas were carefully dissected, rinsed, and stained with DAPI. The retinas were coverslipped with Vectashield and viewed using a Zeiss LSM 700 laser scanning confocal microscope (Carl Zeiss) with a 40× objective. The interval of confocal serial sections was 1 μm and 10–15 serial sections were acquired per retina sample. The cells could be continuously counted from one section to the next, as each section overlapped. The sampled areas were measured along the nasotemporal and dorsoventral axes. One sampled area represented 160 × 160 μm^2^. Neurons in each field were counted (80–95 cells per field); endothelial cells and astrocytes were excluded from the count. Endothelial cells were clearly identified by their elongated shape and close association with blood vessels. Astrocytes were identified by their small cell bodies with dense nuclei and slight displacement toward the optic fiber layer. We sampled 26 or 28 areas in each retina. The sampled areas represented 0.67 or 0.72 mm^2^. The total number of neurons was obtained by multiplying the sampled density by the total retinal area (measured using the Zeiss AxioCam HR digital camera). The total area of each retina was approximately 3.51 mm^2^ and there were approximately 12,254 neurons in the GCL.

## Results

### Morphological characteristics of melanopsin-IR cells

**Fig 2:**
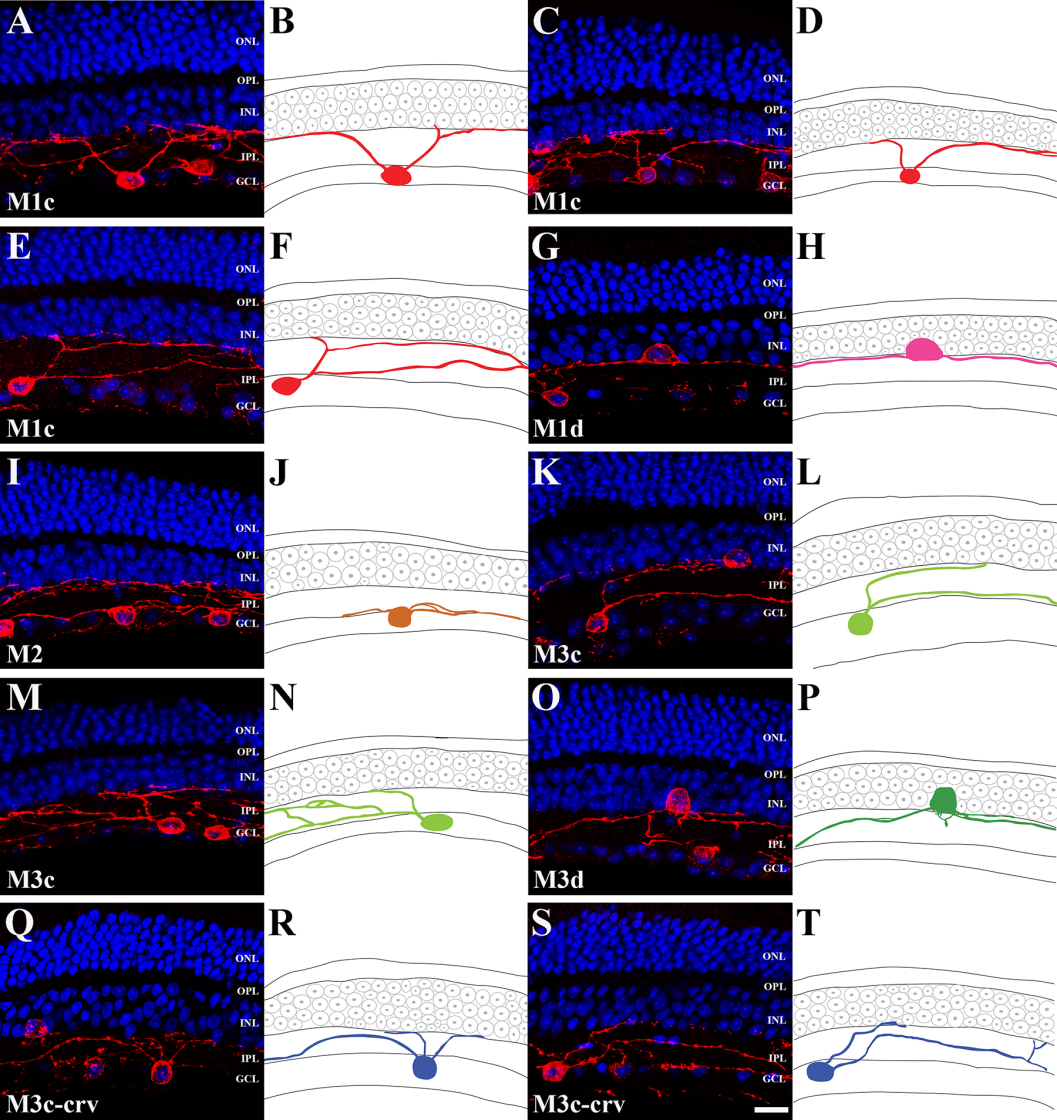
Melanopsin-IR cells in 50 μm vertical sections of *R*. *ferrumequinum* retina. Melanopsin-IR cell types were revealed through stacked confocal images and drawings. (A-F) The M1c type showed monostratified dendritic arborization at the uppermost OFF sublayer of the IPL. M1c cell bodies lay in the GCL. (G-H) M1 displaced RGCs. The M1d type also displayed monostratified dendritic arborization at the uppermost OFF sublayer of the IPL and cell bodies lay in the INL. (I-J) The M2 type showed monostratified dendritic arborization at the bottommost ON sublayer of the IPL, M2 cell bodies lay in the GCL. (K-N) The M3c type displayed dendritic arborization at both the ON and OFF sublayers of the IPL. The M3c cell bodies lay in the GCL. (O-P) The M3d type had bistratified dendritic arborization and cell bodies in the INL. (Q-T) The M3c-crv type had a curved dendrite and cell bodies in the GCL. (A, C, E, G, I, K, M, O, Q, S) Fluorescence photomicrographs. (B, D, F, H, J, L, N, P, R, T) Drawings of melanopsin-IR cells, distinguished by different colors; red (M1c), pink (M1d), brown (M2), yellow-green (M3c), green (M3d), blue (M3c-crv). IR, immunoreactive; RGC, retinal ganglion cell; IPL, inner plexiform layer; GCL, ganglion cell layer; INL, inner nuclear layer; OPL, outer plexiform layer; ONL, outer nuclear layer. Scale bar = 10 μm.

In previous studies, melanopsin-IR cells have been classified into several subtypes based on their dendritic stratifications and soma locations [20, 41, 43]. Fig 2 demonstrates each melanopsin-IR cell type via the vertical sections. Three types of melanopsin-IR cells were found in *R*. *ferrumequinum*: M1 type, M2 type and M3 type. The cell types were decided based on the morphology of melanopsin-IR cells identified previously in mice [15, 20] and rats [41, 44]. Cells were classified according to the location of the cell body as follows: cell bodies conventionally located in the GCL (conventional cells; M1c and M3c) and cell bodies displaced in the INL (displaced cells; M1d and M3d). Drawings of the melanopsin-IR cells were added next to the stacked confocal images of each cell type to accurately represent each cell. Fig 2A–2F show M1c type cells with soma conventionally located in the GCL and dendrites from perikaryon stretching into the top of the OFF sublayer of the IPL. Fig 2G and 2H show M1d type cells with displaced soma in the INL and terminal dendrites reaching the top of the OFF sublayer of the IPL. Fig 2I and 2J show M2 type cells with soma located in the GCL and dendrites reaching the bottom of the ON sublayer of the IPL. Fig 2K–2N show bistratified M3c type cells with soma located in the GCL and terminal dendrites stretching into both the ON and OFF sublayers of the IPL. Fig 2O and 2P illustrate M3d type cells with displaced soma in the INL and terminal dendrites from perikaryon reaching both the ON and OFF sublayers of the IPL. Fig 2Q–2T show two M3c-crv (curved) type cells with soma located in the GCL and a curved dendrite leading up toward the OFF sublayer of the IPL and down to the ON sublayer of the IPL. We designated these cells as M3c-crv in the present study as we think these cells are a variation of the M3c. In the drawings, each cell type was shown in a different color as follows: red for the M1c, pink for the M1d, brown for the M2, yellow-green for the M3c, green for M3d, and blue for the M3c-crv type.

**Fig 3:**
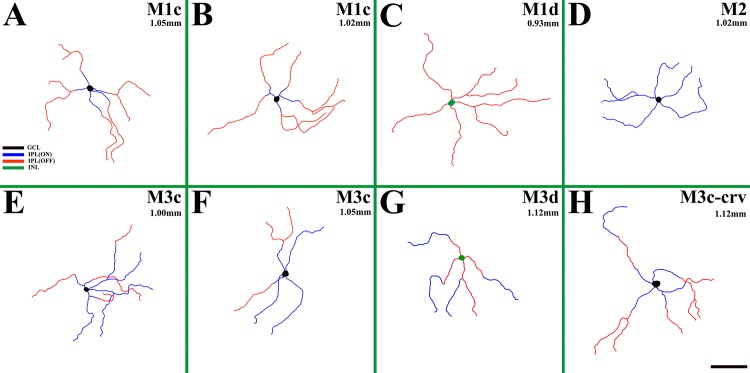
Drawings of melanopsin-IR cells somato-dendritic profiles. The colors used differently for each stratum is as follows: black (GCL), blue (ON sublayer of the IPL), red (OFF sublayer of the IPL), and green (INL). (A and B) Tracing drawings of the M1c type. (C) A tracing drawing of the M1d type. (D) A tracing drawing of the M2 type. (E and F) Tracing drawings of the M3c type. (G) A tracing drawing of the M3d type. (H) A tracing drawing of the M3c-crv type. IR, immunoreactive; RGC, retinal ganglion cell; IPL, inner plexiform layer; GCL, ganglion cell layer; INL, inner nuclear layer. Scale bar = 50 μm.

**Fig 4:**
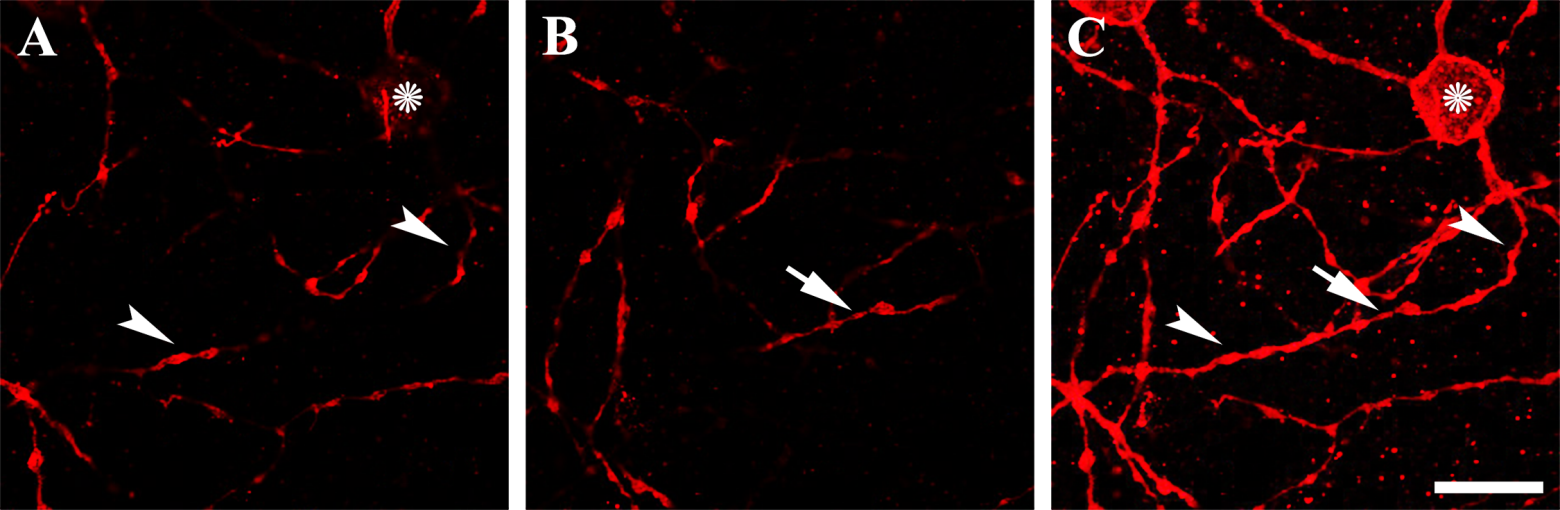
Descriptions of M3c-crv type of ipRGCs on confocal images. (A) Two white arrowheads indicate the dendrite located in the ON sublayer of the IPL. The cell body in the GCL is highlighted by the asterisk. (B) The white arrow shows the dendrite located in the OFF sublayer of the IPL. (C) The stacked confocal image. IPL, inner plexiform layer; GCL, ganglion cell layer. Scale bar = 20 μm.

Tracing drawings of the melanopsin-IR cell types (i.e., M1c, M1d, M2, M3c, M3d and M3c-crv) and somato-dendritic profiles identified in whole-mount *R*. *ferrumequinum* retinas are shown in Fig 3. Cells were identified and colored according to their stratum as follows: black (GCL), blue (ON sublayer of the IPL), red (OFF sublayer of the IPL), and green (INL). Retinal eccentricities are indicated at the top of each drawing. As in the above description, cell bodies of M1 type lay either in the GCL (Fig 3A and 3B) or INL (Fig 3C) and the terminal dendrites were localized in the OFF sublayer of the IPL. Both the M1c type and M1d type had medium dendritic fields with sparsely branched dendrites (Fig 3A–3C). The cell body of M2 type lay in the GCL and the dendrites were observed in the ON sublayer of the IPL. The M1 type had sparse dendrites, whilst M2 type had a medium dendritic field with less bushy dendrites than the M1 type (Fig 3D). Cell bodies of the M3 type lay either in the GCL (Fig 3E and 3F) or INL (Fig 3G), and dendrites were found both in the ON and OFF sublayers of the IPL. Both M3c and M3d types had medium dendritic fields with sparse dendrites (Fig 3E–3G). The cell body of the M3c-crv type lay in the GCL with a curved dendrite leading up to the OFF sublayer of the IPL and then down to the ON sublayer of the IPL. The other dendrites of the M3c-crv type stretched toward the OFF sublayer of the IPL. The M3c-crv type also displayed a medium dendritic field with sparse dendrites (Fig 3H). In general, M1, M2 and M3 types had 2–5 primary dendrites. M3c-crv type melanopsin-IR cells have not yet been observed in any other animals except humans [45]. The curved structure of the M3c-crv type dendrite is seen across two focal planes in the ON and OFF sublayers of the IPL (Fig 4A and 4B). In Fig 4A, two white arrowheads emphasize the dendrite located in the ON sublayer of the IPL, while the asterisk indicates the cell body shown faintly. In Fig 4B, a white arrow shows the dendrite located in the OFF sublayer of the IPL. Fig 4C is the stacked confocal image of the M3c-type cell.

**Fig 5:**
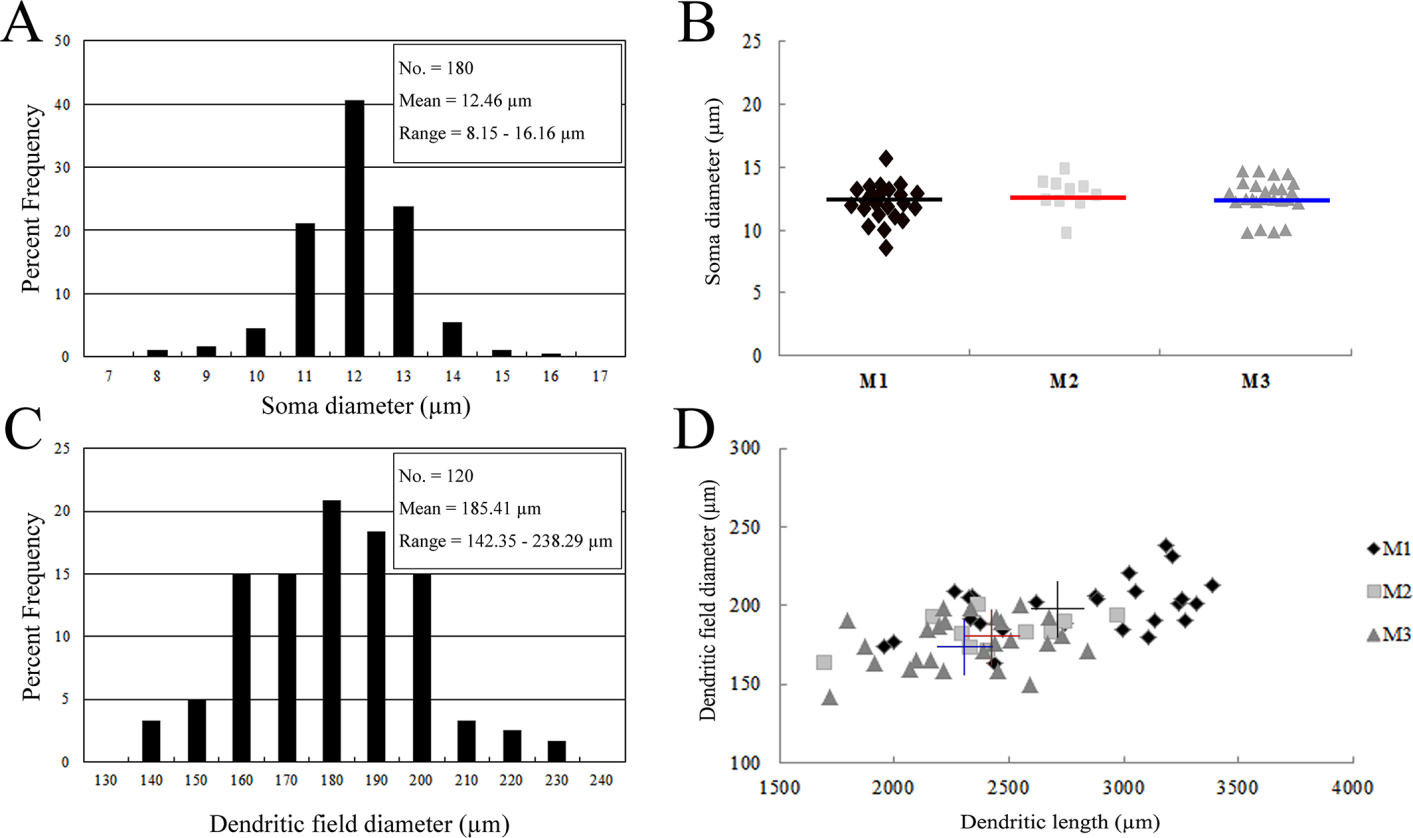
Histogram of the soma and dendritic field diameters of melanopsin-IR cells and differences in soma, dendritic field diameter and dendritic length of the M1, M2 and M3 types in the *R*. *ferrumequinum* retina. The fields were positioned in the mid-peripheral retina and the best labeled neurons were analyzed. (A) A total of 180 neurons labeled with melanopsin were measured in the three retinas. The soma diameter ranged from 8.15 to 16.16 μm. (B) Soma diameters of the different cell types were measured in the three retinas. The different cell types were marked using different shapes as follows: a diamond-square for the M1 type, square for the M2 type, and triangle for the M3 type. The lines represent the mean soma diameter. (C) A total of 120 neurons labeled with melanopsin were measured for the three retinas. The dendritic field diameter ranged from 142.35 to 238.29 μm. (D) The dendritic field diameters and dendritic lengths of the different cell types were measured in the three retinas. The different cells types have been marked by the following shapes: the diamond-square for the M1 type, square for the M2 type, and triangle for the M3 type. The crosses with different colors represent the mean dendritic field diameters and dendritic lengths. IR, immunoreactive.

The melanopsin-IR cells in the *R*. *ferrumequinum* retina had medium cell bodies and dendritic field diameters (Fig 5). The soma diameter was measured for 180 neurons in three retinas. The mean soma diameter was 12.46 ± 1.15 μm (mean ± standard deviation [SD]; n = 180), which ranged 8.15–16.16 μm (Fig 5A). The soma diameter by three cell types was measured for 60 neurons in three retinas, i.e., 25 M1 type neurons, 10 M2 type neurons and 25 M3 type neurons. Different shapes by cell types were marked (i.e., diamond-square for the M1 type, square for the M2 type, and triangle for the M3 type). Different colored lines were used for the different cell types and represented the mean soma diameter. The mean soma diameters of the M1, M2, and M3 types were 12.39 ± 1.69 μm (n = 25, p < 0.05, ANOVA), 13.08 ± 1.38 μm (n = 10, p < 0.05, ANOVA), and 12.72 ± 1.57 μm (n = 25, p < 0.05, ANOVA), respectively. There were no significant differences among the three cell types (Fig 5B). The dendritic field diameter was measured for 120 neurons in the three retinas. The mean dendritic field diameter was 185.41 ± 18.72 μm (n = 120), which ranged 142.35–238.29 μm (Fig 5C). The dendritic diameters and lengths of the cell types are shown in Fig 5D. The different cells types have been marked with the following shapes: diamond-square for the M1 type, square for the M2 type, and triangle for the M3 type. Different colored crosses were used for the average dendritic field diameter and length as follows: black for the M1 type, red for the M2 type, and blue for the M3 type. The mean dendritic field diameter and dendritic length of the M1 type were 198.84 ± 17.25 μm (n = 25, p < 0.05, ANOVA) and 2791.44 ± 441.08 μm (n = 25, p < 0.05, ANOVA), respectively. The mean dendritic field diameter and the mean dendritic length of the M2 type were 183.26 ± 11.40 μm (n = 10, p < 0.05, ANOVA) and 2425.71 ± 352.79 μm (n = 10, p < 0.05, ANOVA), respectively. The mean dendritic field diameter and mean dendritic length of the M3 type were 176.74 ± 15.97 μm (n = 25, p < 0.05, ANOVA) and 2306.81 ± 299.02 μm (n = 25, p < 0.05, ANOVA), respectively (Fig 5D).

### Distribution analysis of melanopsin-IR RGCs

**Fig 6:**
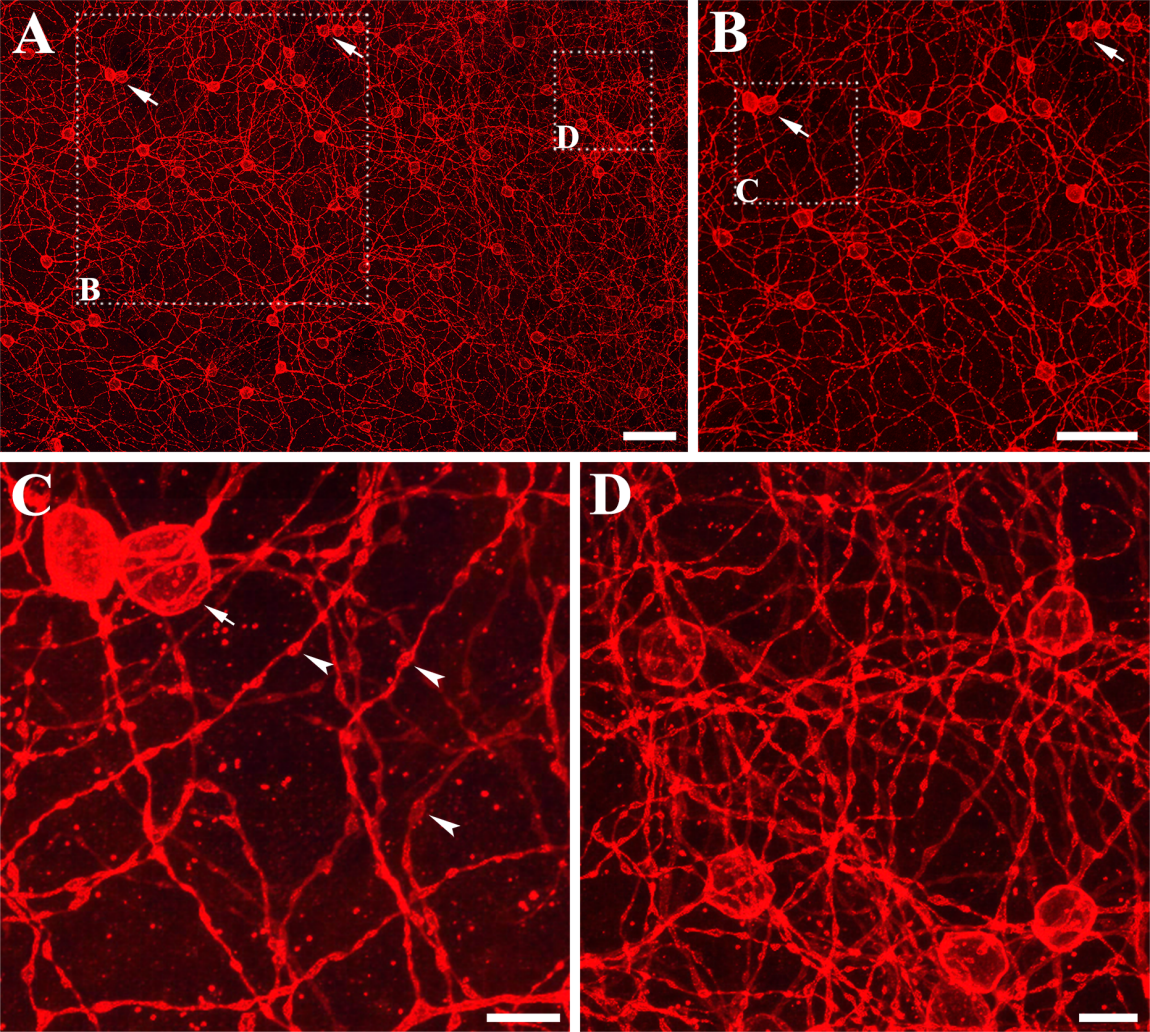
Distribution patterns of melanopsin-IR cells in the *R*. *ferrumequinum* retina. Confocal images were acquired as z-stacks from a mid-peripheral area in the *R*. *ferrumequinum* retina. (A-D) Each image was obtained at different magnifications. (A-C) Melanopsin-IR cells were not evenly distributed in the retina; complex dendrites were entangled with one another. Arrows show closely positioned cell bodies. (B) Medium magnification photomicrograph of the inset square of (A) clearly shows the positions of the cell bodies. (C) The varicosities along the sparsely branched dendrites of the whole-mount retina are indicated by arrow heads. (D) A dendritic complexity is shown in one location of the whole-mount retina. The curving and stretching processes and the multiple subdivided dendritic branches are displayed. IR, immunoreactive. (A and B) Scale bar = 50 μm, (C and D) Scale bar = 10 μm.

Fig 6 shows the distribution patterns of melanopsin-IR cells on a whole-mount retina. The stacked confocal images were acquired from mid-peripheral areas, with a 40× objective. In the *R*. *ferrumequinum* retina, a considerable number of melanopsin-IR cells covered the whole retina. Their dendrites were complex and of a tangled structure. In addition, the melanopsin-IR cells displayed curving and stretching processes and had multiple branches that subdivided from the main branches. Melanopsin-IR cells were not evenly distributed in the retina (Fig 6), with two or three cell bodies positioned closely to other cells (Fig 6A–6C, cell bodies were shown by arrows). Fig 6B shows a medium magnification photomicrograph of the inset square in Fig 6A to clearly demonstrate the cell body positions. Fig 6C shows the magnified area of one location in Fig 6B indicating two closely positioned cell bodies and the dendrites with varicosities along the arbors. The three arrowheads in Fig 6C indicate round/oval-shaped varicosities. Fig 6D shows the dendritic complexity in a higher magnification than the inset square in Fig 6A. The complex processes covered the retina, with many overlapped and entangled processes.

The total number of melanopsin-IR cells was counted in four retinas. We observed a high density of melanopsin-IR cells (214.29–242.03 cells/mm^2^) in the *R*. *ferrumequinum* retinas. The mean melanopsin-IR cell density was 227.12 ± 11.41 cells/mm^2^ (n = 4). The total number of melanopsin-IR cells was measured in each retina. A total of 16 and 37 areas were sampled and the total cell counts were estimated for the areas along the central dorsoventral and nasotemporal axes. Retina #1, #2, #3, and #4 contained a total of 754.30 cells, 815.65 cells, 828.00 cells, and 881.00 cells, respectively. The mean total number of melanopsin-IR cells was 819.74 ± 52.03 cells/retina (Table 1).

**Table 1:** The density of melanopsin-IR RGCs in microbat, *R*. *ferrumequinum*.

Retina	Sampled area (*n*)	Sampled area* (mm^2^)	Neurons counted	Mean density (cells/mm^2^)	Total retina area (mm^2^)	Total melanopsin-IR neurons
Retina #1	16	1.54	330	214.29	3.52	754.30
Retina #2	16	1.54	347	225.32	3.62	815.65
Retina #3	37	3.56	828	226.85	3.57	828.00
Retina #4	37	3.56	881	242.03	3.63	881.00
Mean±SD				227.12±11.41	3.59 ±0.05	819.74±52.03

* One sampled area: 310 × 310μm^2^. IR, immunoreactive; RGC, retinal ganglion cell; SD, standard deviation.

**Fig 7:**
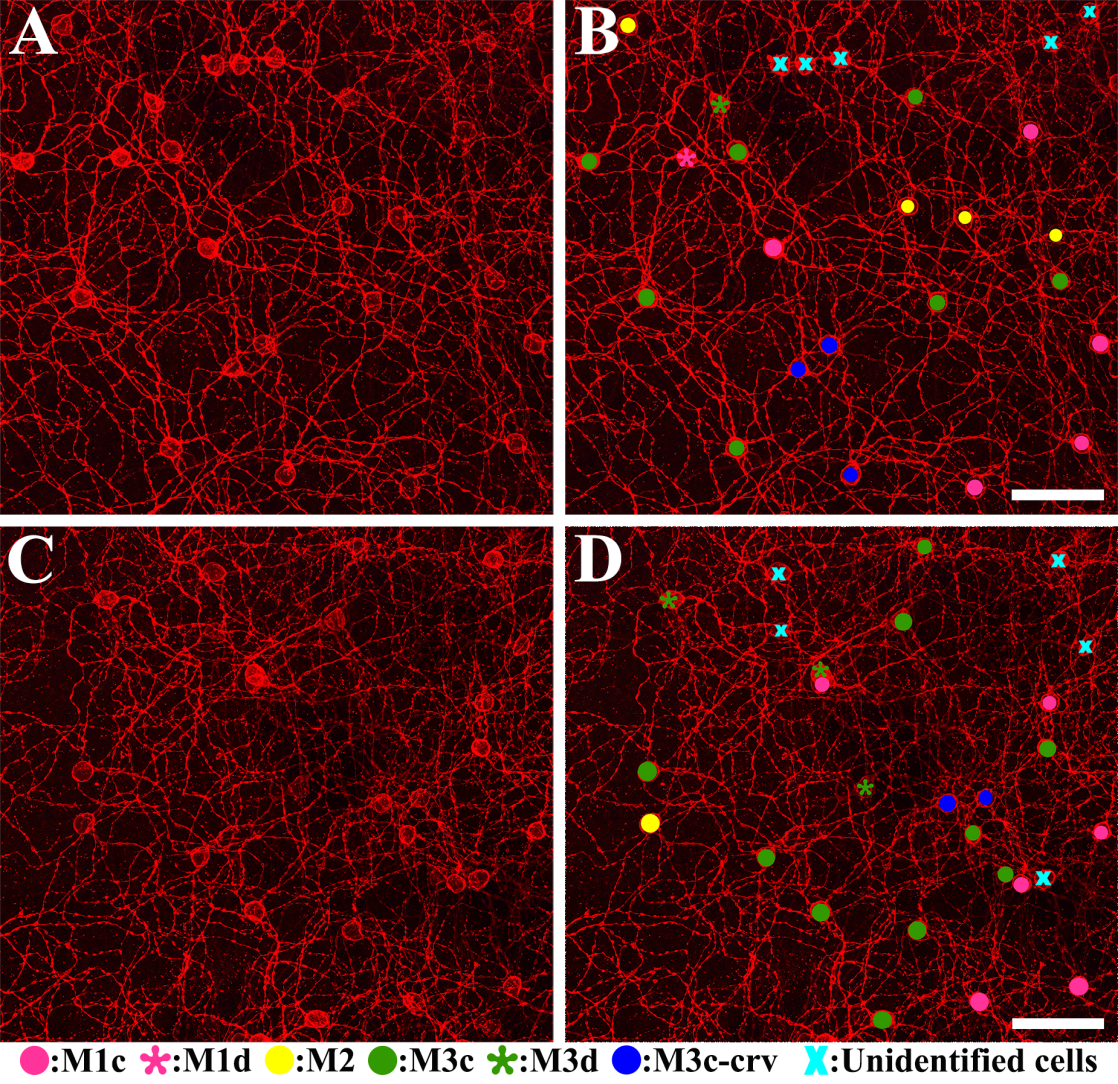
Distribution of each melanopsin-IR cell type on whole-mount retinas. Cell types are represented by different colors and symbols as follows: pink circle (M1c); pink asterisk (M1d); yellow circle (M2); green circle (M3c); green asterisk (M3d); blue circle (M3c-crv); and a sky-blue X (unidentified cells). Two different retinas were used to show the distribution of cell types. (A, B) and (C, D) show the same areas in different retinas. (B, D) Display the whole-mount retina showing the different cell types. IR, immunoreactive. Scale bar = 50 μm.

Fig 7 shows the distribution of each melanopsin-IR cell type on whole-mount retinas. Fig 7A–7D show the same areas of the two different mid-peripheral retinas. Fig 7B and 7D show analytical maps of each type of melanopsin-IR cells from Fig 7A and 7C, respectively. Different colors and shapes were used to represent different types of melanopsin-IR cells as follows: a pink circle for M1c, a pink asterisk for M1d, a yellow circle for M2, a green circle for M3c, a green asterisk for M3d, a blue circle for M3c-crv, and a sky-blue X for unidentified cells. In retina 1: 5 M1c type, 1 M1d type, 4 M2 type, 7 M3c type, 1 M3d type, 3 M3c-crv type and 5 unidentified cells were observed (Fig 7B). In retina 2: 6 M1c type, 1 M2 type, 10 M3c type, 3 M3d type, 2 M3c-crv type and 5 unidentified cells were counted (Fig 7D). The percentage of each cell type is indicated in Table 2. We sampled 11 areas in the mid-peripheral part of each retina and estimated the percentage of each cell type in the stacked confocal images. Cell types were counted from three whole-mount retinas and each proportion varied from 4.69 ± 0.24 to 26.66 ± 1.03 (%). The percentage of each cell type was as follows: 21.00 ± 0.67 (%; M1c); 5.15 ± 1.36 (%; M1d); 5.79 ± 1.27 (%; M2); 26.66 ± 1.03 (%; M3c); 4.69 ± 0.24 (%; M3d); 7.67 ± 0.58 (%; M3c-crv); and 29.38 ± 2.49 (%; unidentified cells) (Table 2). The M3c type represented the greatest percentage of melanopsin-IR cells and M3d type represented the lowest percentage. Although these results are limited due to the small number of samples, they provide important information on the percentage of each melanopsin-IR cell type in the *R*. *ferrumequinum* retina.

**Table 2:** Density of each melanopsin-IR cell type in the *R*. *ferrumequinum* retina.

Retina	Sampled area (*n*)	Sampled area* (mm^2^)	Total retina area (mm^2^)	No. of M1c cells counted	Total M1c (%)	No. of M1d cells counted	Total M1d (%)	No. of M2 cells counted	Total M2 (%)	No. of M3c cells counted	Total M3c (%)	No. of M3d cells counted	Total M3d (%)	No. of M3c-crv cells counted	Total M3c-crv(%)	No. of unidentified cells counted	Total unidentified cells (%)	Total no. of cells counted
Retina #1	11	0.83	3.52	18	21.69	3	3.61	6	7.23	23	27.71	4	4.82	7	8.43	22	26.51	83
Retina #2	11	0.83	3.57	26	20.97	7	5.65	6	4.84	33	26.61	6	4.84	8	6.45	38	30.65	124
Retina #3	11	0.83	3.62	23	20.35	7	6.19	6	5.31	29	25.66	5	4.42	8	7.08	35	30.97	113
Mean±SD					21.00±0.67		5.15±1.36		5.79±1.27		26.66±1.03		4.69±0.24		7.67±0.58		29.38±2.49	

* One sampled area: 250 × 300 μm^2^. IR, immunoreactive; SD, standard deviation.

### Cell densities of ganglion cells, displaced amacrine cells, and melanopsin-IR RGCs

The GCL consists of RGCs and displaced amacrine cells. To identify the total number of neurons in the GCL, confocal microscopy was used to image cells stained with DAPI in three retinal whole-mounts. Neurons were counted in each of the 26–28 sampled areas from both the dorsoventral and nasotemporal central meridians. Non-neural cells, i.e., endothelial cells and astrocytes, were not counted. Endothelial cells were identified by their elongated shape and blood vessels, and astrocytes were displaced toward the optic fiber layer and possessed small cell bodies with dense nuclei. The total number of neurons in the GCL was measured by multiplying the average density of cells in each retina by the total area of the retina. The density of neurons in the GCL ranged from 3,189.17 to 3,702.57 cells/mm^2^. The mean density was 3,497.17 ± 271.64 cells/mm^2^ (n = 3). The total number of neurons in the GCL ranged from 11,544.81 to 12,851.14 cells/retina. The mean total number of neurons in the GCL was 12,254.17 ± 660.39 cells/retina (n = 3) (Table 3A).

**Table 3:** The total number of neurons in the GCL and total optic nerve axons in the *R*. *ferrumequinum*.

A. Ganglion cell layer neurons					
Retina	Neurons counted	Sampled area(mm^2^)	Mean density (cells/mm^2^)	Total retina area (mm^2^)	Total neurons in GCL
Retina #1	2,654	0.7168	3,702.57	3.34	12,366.57
Retina #2	2,286	0.7168	3,189.17	3.62	11,544.81
Retina #3	2,396	0.6656	3,599.76	3.57	12,851.14
Mean±SD	2,445±188.90	0.6997±0.0296	3,497.17±271.64	3.51±0.15	12,254.17±660.39
B. Optic nerve axons					
Optic nerve	Axons counted	Sampled area (μm^2^)	Mean density (axons/μm^2^)	Total nerve area(μm^2^)	Total axons
Optic nerve #1	1,656	4356.964	0.380081	13842.88	5,261.42
Optic nerve #2	1,865	4356.964	0.428050	12459.37	5,333.23
Optic nerve #3	1,691	4356.964	0.388114	12734.59	4,942.47
Mean±SD	1,737±111.939		0.39875±0.0256	13012.28±732.365	5,179.04±208.00

GCL, ganglion cell layer; SD, standard deviation.

**Fig 8:**
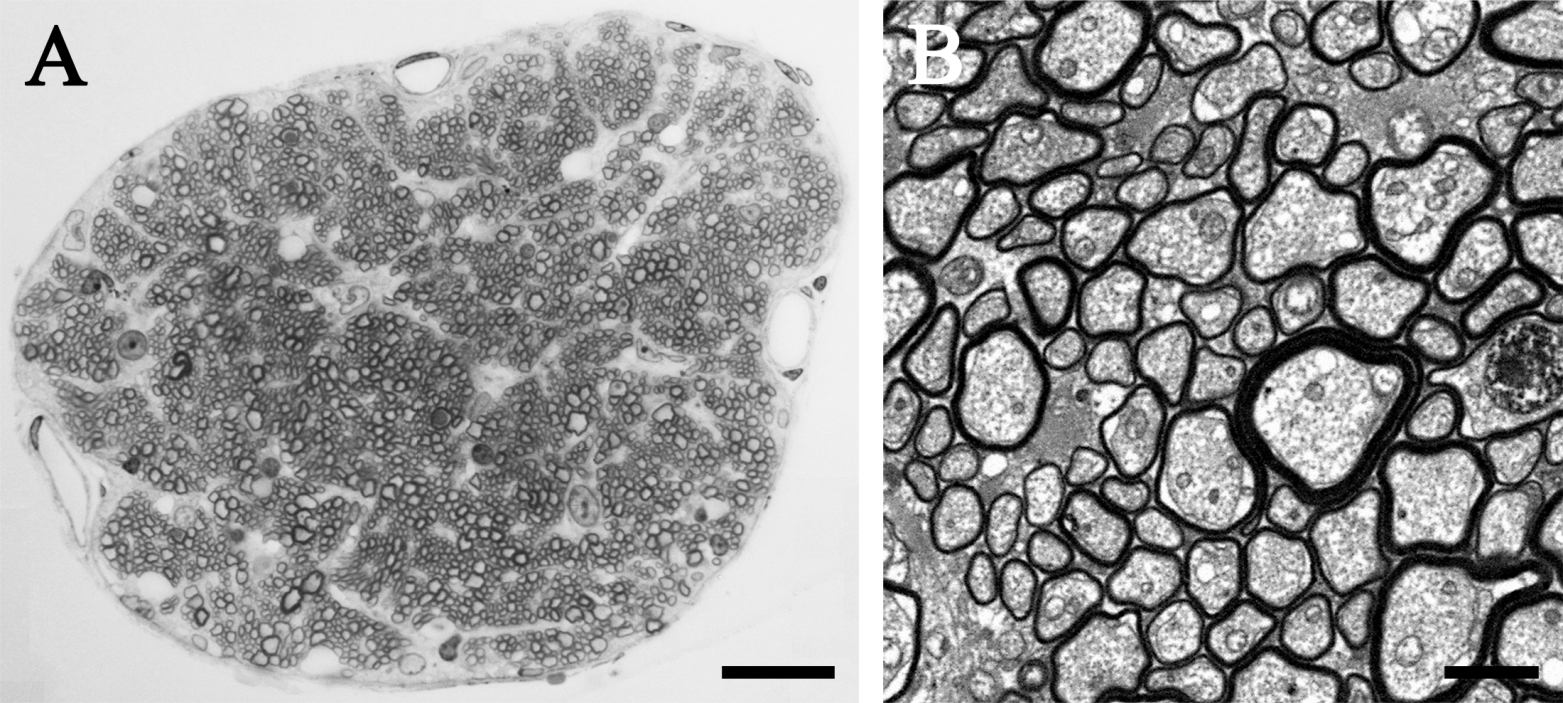
Electron micrograph from sections of *R*. *ferrumequinum* optic nerve. Large and small myelinated axons are shown. The two images were observed at different magnifications: (A) 100× objective; (B) 700× direct mag, 6680× print mag. The whole optic nerve is shown. (A) Scale bar = 20 μm. (B) Scale bar = 2 μm.

RGCs are neurons that are found in the GCL. The total number of RGCs was identified by counting the number of axons in the three optic nerves (Fig 8A and 8B), at ~300 μm from the posterior pole of the eye. Most axons in the optic nerve are myelinated and can thus, be easily identified with electron microscopy. The total number of optic nerve fibers ranged 4,942.47–5,333.23 fibers/retina (representing 4,942.47–5,333.23 RGCs). On average, the total number of optic nerve fibers was 5,179.04 ± 208.00 fibers/retina (n = 3) (Table 3B). As there were 12,254.17 ± 660.39 neurons in the GCL and 5,179.04 ± 208.00 RGCs, the RGCs represented 42.26% of the total cell number. The total number of melanopsin-IR cells was 819.74 ± 52.03 cells/retina. Therefore, melanopsin-IR cells accounted for 15.83% of RGCs in the microbat retina (including displaced cells).

## Discussion

Mammals have multiple types of melanopsin-IR cells in the retina. Based on cell body locations and dendritic stratification patterns, for example, there are two types of melanopsin-IR cells in humans, marmosets and macaques; i.e., the outer-stratifying cells and inner-stratifying cells [17, 43]. At least five types of melanopsin-IR cells (M1-M5) have been identified in the mouse and rat retinas [3, 19, 20, 46], while two types of melanopsin-IR cells (M1 and M3) have been identified in the Mongolian gerbil retina [47]. In the present study, we identified three types of melanopsin-IR cells in the *R*. *ferrumequinum* retina (M1, M2 and M3 types). However, other types of melanopsin-IR cells might be identifiable in the microbat retina if transgenic or single cell injection techniques, which have been used in rodents to identify M4 and M5 subtypes, are applied. The morphological characteristics of melanopsin-IR cells of the M1 type through to the M3 type corresponded to those seen in previous studies on other animals [20, 23, 48]. The M3c-crv type had a cell body in the GCL and curved dendrites leading from the perikaryon to the OFF sublayer of the IPL, which then proceeded down to the ON sublayer of the IPL. Although the presence of ipRGCs with a curved dendrite in both the ON and OFF sublayers of the IPL has not been reported, a recent report studying human ipRGCs (Fig 3) [45] demonstrated this type of RGCs. M4 and M5 types, which make up a very low population of ipRGCs in mouse and rat [3, 19, 46], have not been found in the bat retina. Taken together, the evidence suggests that there are some subtle discrepancies between the species with regard to ipRGCs types. The variability of melanopsin-IR cells between different species is still unknown.

The proportion of each type of melanopsin-IR cell varies among animals. In humans, outer-stratifying cells represent 60% and inner-stratifying cells represent 40% of melanopsin-IR cells [43]. In the marmoset, outer-stratifying and inner-stratifying cells represent 86% and 14% of melanopsin-IR cells, respectively, while these figures are 90% and 10%, respectively, for the macaque [17]. In contrast, a recent study suggested that outer-stratifying cells represent 61% and inner-stratifying cells represent 39% of melanopsin-IR cells in the macaque [49]. In the rodent retina, the following percentages were reported: M1 type (22–67.5%), M2 type (25–52%) and M3 type (7.5–26%) in mouse [20, 21, 23, 39, 40, 48]; and M1 type (74%) and M2 type (~25%) in the rat [41]. M3, M4 and M5 type cells constitute a very small proportion of ipRGCs seen in the mouse and rat [3, 19, 20, 40, 46]. The Mongolian gerbil retina contains M1 type (97.43%) and M3 type (2.56%) cells without the presence of M2 type [47]. These previous results suggest that M1 and M2 types are the dominant types in most animals studied thus far and that M3 type contributes to only a small percent of ipRGCs among other animals [20, 40, 47]. Our study demonstrated that M1 type (26.15%), M2 type (5.79%), and M3 type (39.02%) all exist in the *R*. *ferrumequinum* retina. Thus, in contrast to the previous studies, the present study showed that the M3 type is the most dominant melanopsin-IR cell type in the *R*. *ferrumequinum* retina.

The ipRGCs innervate several brain regions. The suprachiasmatic nucleus (SCN) is the principle region that ipRGCs axons project to. It is involved in the moderation of circadian rhythms. Since ipRGCs also project to the ventrolateral preoptic nucleus (VLPO), the olivary pretectal nucleus (OPN) and the pretectal area (PTA), ipRGCs may control sleep and wake states and the pupillary reflex [9, 12, 18, 48]. In addition, other studies show that ipRGCs also project to the lateral geniculate nucleus (LGN) and the superior colliculus (SC). The LGN contributes to the processing of visual information, while the SC helps to direct eye movements in response to visual clues [10, 14–16, 43, 50]. Some research has demonstrated that different subtypes of ipRGCs innervate different brain regions. For example, in the mouse, the SCN is innervated by M1 type (80%) and M2 type (20%), and the OPN is innervated by M1 type (45%, mainly shell region of the OPN) and M2 type (55%, mainly core region of the OPN) [6]. Consequently, the M1 type of melanopsin-IR cells is the dominant type that projects to the SCN; therefore, it may largely participate in the control of circadian rhythms. Further, a genetic ablation study of Brn3b-expressing ipRGCs demonstrated that a subpopulation of M1 ipRGCs (Brn3b+) innervating the OPN plays an important role in control of pupillary light reflex [4, 51]. Although the M2 type strongly innervates the OPN core [4, 6], little functional information has been assigned to this area [4, 52]. Thus, as the M1 type and M2 type account for 26.15% and 5.79% of melanopsin-IR cells in the *R*. *ferrumequinum* retina, respectively, the M1 type may primarily participate in the control of circadian rhythms and pupillary reflex. Since the M3 type is known to innervate the SC, it may therefore be involved in directing eye movement [19]. Thus, the high proportion of the M3 type (39.02%) seen in the *R*. *ferrumequinum* retina suggests that these cells may play a crucial role in the microbat, for the direction of eye movements in response to visual clues.

Although the soma and dendritic field diameters of ipRGCs vary among animals, the soma size of ipRGCs is generally small to medium [20, 40, 41], while the dendritic field diameters of ipRGCs are usually large [15, 20, 23]. In the *R*. *ferrumequinum* retina, the mean soma diameter was 12.46 ± 1.15 μm (8.15–16.16 μm). The mean dendritic field dimeter and mean dendritic length were 185.41 ± 18.72 μm (142.35–238.29 μm) and 2507.99 ± 431.75 μm (1698.37–3384.26 μm), respectively. In humans, for example, the mean soma diameters were 19 μm for outer-stratifying cells and 23 μm for inner-stratifying cells [49], while it was 14.8 ± 1.1 μm and 17.0 ± 3.8 μm, respectively, in marmoset [17] and 14 μm and 16 μm, respectively, in macaques [49]. Further, the mean soma diameters in some other species are as follows: 12.2–17.0 μm for M1 type, 14.8–21.8 μm for M2 type, 14.5–17.8 μm for M3 type and 20.1–31.47 μm for M4 type in mice [15, 20, 22–24, 39, 40, 53]; 12.8 ± 0.4 μm for M1 type, 15.1 ± 0.9 μm for M2 type, 14.8 ± 0.8 μm for M3 type, 22.2 ± 0.7 μm for M4 type and 15.3 ± 0.5 μm for M5 type in rats [3]; 10.61 ± 1.35 μm in Mongolian gerbils [47]; 14.0 ± 1.6 μm in cats [54]; and 12.2 ± 0.4 μm in mole rats [55]. Moreover, the mean dendritic field diameters, for example, were 690 ± 148 μm for outer-stratifying cells and 646 ± 151 μm for inner-stratifying cells in the human retina [49]; 520.7 ± 133.1 μm for outer-stratifying cells and 593.1 ± 85.5 μm for inner-stratifying cells in marmosets [17], 718 ± 220 μm for outer-stratifying cells and 761 ± 216 μm for inner-stratifying cells in macaques [49]; 275–377 μm for M1 type, 314–422.9 μm for M2 type, 449–477 μm for M3 type and 301–359.6 μm for M4 type in mice [15, 20, 22–24, 40]; 543.3 ± 24.4 μm for M1 type, 426.4 ± 24.8 μm for M2 type, 382.2 ± 68.1 μm for M3 type, 409.7 ± 26.7 μm for M4 type and 284.8 ± 11.6 μm for M5 type in rats [3]; 219.95 ± 25.93 μm in Mongolian gerbils [47] and approximately 303 μm in mole rats (calculated dendritic field diameter from their dendritic field area data, [55]). The mean dendritic lengths were 1605.0–2350.1 μm for M1 type, 1553.0–4603.6 μm for M2 type, 4441.2 ± 331.4 μm for M3 type and 4584.0–4751.0 μm for M4 type in mice [15, 22–24]. The combined results demonstrated that melanopsin-IR cells in the *R*. *ferrumequinum* retina had a medium soma diameter, medium dendritic field diameter, and small dendritic length. Although there were no significant differences in the soma diameter among cell types, the M2 type had a little larger soma diameter than the other types in the microbat. This result is accordant with the previous results [15, 20, 22, 24, 39, 40, 41]. The results also show that, until now, there is no direct relationship linking animal size to the size of melanopsin-IR cells.

The density of melanopsin-IR cells in the retina is very low [16, 18, 20, 43, 46, 47, 49, 54, 56, 57]. For example, the densities of ipRGCs were 3–5 cells/mm^2^ in periphery and 20–25 cells/mm^2^ in parafoveal human retina [43]. Another study reported, 20–40 cells/mm^2^ at about 2 mm eccentricity and ~10 cells/mm^2^ at about 8 mm eccentricity in the human retina [45]. Further, the ipRGC density was also reported to be 18 cells/mm^2^ in the nasal and inferior pieces of the marmoset retina, 10 cells/mm^2^ in the nasal and inferior pieces of macaque retina [17], and 18.33 cells/mm^2^ in Mongolian gerbils [47]. Previous studies have also demonstrated that melanopsin-IR cells represent a very small subset of RGCs, which comprise approximately 0.2–0.4% in the human [43, 49], less than 0.2% in the marmoset [57], 0.5% in the Mongolian gerbil [47], approximately 1–2% in the mouse [20, 46], 2.5% in the rat [18], ~1.5% in the hamster [16, 56], and 1% in the cat [54]. In our previous study, we proposed that nocturnal animals may have a tendency towards having a higher density and proportion of ipRGCs than diurnal animals [47]. Nocturnal animals, such as the mouse, rat, hamster, and cat have a slightly larger proportion of melanopsin-IR cells than diurnal animals, such as the human, marmoset, and Mongolian gerbil. The total number of neurons in the GCL of the *R*. *ferrumequinum* retina was 12,254.17 ± 660.39. The total number of RGCs was 5,179.04 ± 208.00. Thus, RGCs make up around 42.26% of total neurons in the GCL of the *R*. *ferrumequinum* retina. The total number of the RGCs in the present study is accordant with the results of a previous study in another microbat (*Rhinolophus rauxi*, about 4500 [36]) and the proportion of the RGCs in retina was similar to various mouse species (41.6% [38], 44% [58], and 40.3% [59]). Thus, based on the results of the present study, we can draw a solid conclusion: the microbat, *R*. *ferrumequinum*, one of the representative nocturnal animals, had a very high density (227.12 ± 11.41 cells/mm^2^) and proportion (15.83%) of ipRGCs in the retina, in comparison to that in other sighted animals (i.e., less than 40 cells/mm^2^ and 1–2% of ipRGCs, respectively). Recently, an interesting study on melanopsin-IR cells in blind mole rat, which has eyes that are completely covered with skin, was reported [55]. According to their study, 87% (788 cells/mm^2^) of the RGCs contained melanopsin. This supports the suggestion that nocturnal animals may have the tendency to have a high density and proportion of melanopsin-IR cells. The different active hours of nocturnal animals might lead to differences in factors such as the availability of food, competition for food, predation, and biological morphology. Thus, it is plausible that different animal lifestyle might be related to the amount of light, which in turn might be an important factor for the different proportion of ipRGCs.

Previous studies have demonstrated that a lack of ipRGCs has effects on non-visual forming system such as circadian rhythms, pupillary light responses, and sleep behaviors [12, 60, 61]. Thus, melanopsin-null mice displayed decreased light-induced phase-shifts in circadian rhythms compared to wild-type mice [60, 62]. In addition, Opn4 -/- mice slept less than wild-type mice and acute light-mediated sleep could not be induced due to lack of melanopsin [61, 63, 64]. These results suggest that the highly enhanced expression of melanopsin may be related to remarkably prolonged sleep behavior in nocturnal animals. However, the underlying reasons for highly-developed ipRGCs in the microbat could not be elucidated with the present anatomical study. Therefore, further physiological and behavioral studies will be needed in the future.

## Conclusions

The present study revealed several important features of melanopsin-IR cells in the *R*. *ferrumequinum* retina. The existence of three types of melanopsin-IR cells (M1 type- M1c and M1d; M2 type; and M3 type- M3c, M3d, M3c-crv) suggests that in the *R*. *ferrumequinum* retina, these cells may contribute to both non-image and image forming vision. In contrast to other animals, M3 type represented the majority of melanopsin-IR cells in the *R*. *ferrumequinum* retina. Melanopsin-IR cell somas were medium-sized and had medium dendritic fields, with 2–5 primary dendrites. The density of melanopsin-IR cells was 227.12 ± 11.41 cells/mm^2^ and constituted 15.83% of the total RGC population. The high density and proportion of melanopsin-IR cells in the microbat, *R*. *ferrumequinum* retina (compared to diurnal animals), may be an important factor in the adaption to a low-light environment.

## Supporting information

S1 File Cell densities of melanopsin-IR cells and neurons in the GCL in microbat, *E*. *serotinus*. (DOCX)

S1 Table The density of melanopsin-IR RGCs in microbat, *E*. *serotinus*. (DOCX)

S2 Table The total number of neurons in the GCL in microbat, *E*. *serotinus*. (DOCX)
