# Supplementary material for: The organization of melanopsin-immunoreactive cells in microbat retina
Source: PLoS One. 2018 Jan 5;13(1):e0190435. doi: 10.1371/journal.pone.0190435 (PMC5755760; doi:10.1371/journal.pone.0190435)
Supplement: S1 File — (DOCX) [file pone.0190435.s001.docx]

**S1 File. Cell densities of melanopsin-IR cells and neurons in the GCL in microbat, *Eptesicus serotinus.***

To verify the present result of a higher density of melanopsin-IR cells in the microbat, we analyzed the total number of melanopsin-IR cells and neurons in the GCL in another species of microbat (*Eptesicus serotinus*) using the same methods as we used for *R. ferrumequinum*. *E. serotinus* bat retinas also had high melanopsin-IR cell densities, which ranged from 369.57 to 395.65 cells/mm^2^. The mean melanopsin-IR cell density was 382.61 ± 13.04 cells/mm^2^ (mean ± standard deviation [SD]; n = 3). There were 909.13 cells in retina #1, 990.96 cells in retina #2, and 929.78 cells in retina #3. The mean total number of melanopsin-IR cells was 943.29 ± 42.55 cells/retina (S1 Table). The densities of the neurons in the GCL ranged from 5,921.79 to 6,966.48 cells/mm^2^. The mean density was 6,370.58 ± 537.66 cells/mm^2^ (n = 3). There were 15,309.72 cells in retina #1, 15,337.43 cells in retina #2, and 16,371.23 cells in retina #3. The mean total number of neurons in the GCL was 15,672.79 ± 605.02 cells/retina (S2 Table). From these results, we estimated the proportion of the melanopsin-IR cells in the *E. serotinus*. Since the ganglion cells in the GCL comprise approximately 40–60% of the total cells in the GCL [13, 28, 53, 59], the proportion of melanopsin-IR cells in another microbat, *E. serotinus*, was approximately 10.03% (where RGC comprise 60% of total cells in the GCL) to 15.05% (where RGC comprise 40% of total cells in the GCL). These results support that microbats have a high density of melanopsin-IR cells.
