# Supplementary material for: The organization of melanopsin-immunoreactive cells in microbat retina
Source: PLoS One. 2018 Jan 5;13(1):e0190435. doi: 10.1371/journal.pone.0190435 (PMC5755760; doi:10.1371/journal.pone.0190435)
Supplement: S1 Table — (DOCX) [file pone.0190435.s002.docx]

| **S1 Table. The density of melanopsin-IR RGCs in microbat, *E. serotinus.*** | | | | | | |
| --- | --- | --- | --- | --- | --- | --- |
| Retina | Sampled  area (*n*) | Sampled  area* (mm^2^) | Neurons  counted | Mean density  (cells/mm^2^) | Total retina area (mm^2^) | Total melanopsin-IR neurons |
| Retina #1 | 9 | 0.23 | 85 | 369.57 | 2.46 | 909.13 |
| Retina #2 | 9 | 0.23 | 88 | 382.61 | 2.59 | 990.96 |
| Retina #3 | 9 | 0.23 | 91 | 395.65 | 2.35 | 929.78 |
| Mean±SD |  |  |  | 382.61 ± 13.04 | - 1. ± 0.12 | 943.29 ± 42.55 |

* One sampled area: 159.9 × 159.9 µm^2^. IR, immunoreactive; RGC, retinal ganglion cell; SD, standard deviation.
