# Supplementary material for: The organization of melanopsin-immunoreactive cells in microbat retina
Source: PLoS One. 2018 Jan 5;13(1):e0190435. doi: 10.1371/journal.pone.0190435 (PMC5755760; doi:10.1371/journal.pone.0190435)
Supplement: S2 Table — (DOCX) [file pone.0190435.s003.docx]

**S2 Table. The total number of neurons in the GCL of microbat, *E. serotinus*.**

| Retina | Neurons  counted | Sampled  area (mm^2^) | Mean density (cells/mm^2^) | Total retina area (mm^2^) | Total neurons in GCL |
| --- | --- | --- | --- | --- | --- |
| Retina #1 | 1,114 | 0.179 | 6,223.46 | 2.46 | 15,309.72 |
| Retina #2 | 1,060 | 0.179 | 5,921.79 | 2.59 | 15,337.43 |
| Retina #3 | 1,247 | 0.179 | 6,966.48 | 2.35 | 16,371.23 |
| Mean ± SD | 1,140.33 ± 96.24 |  | 6,370.58 ± 537.66 | 2.47 ± 0.12 | 15,672.79 ± 605.02 |

GCL, ganglion cell layer; SD, standard deviation.
